# Mechanistic Characterization of Cancer-associated Fibroblast Depletion via an Antibody–Drug Conjugate Targeting Fibroblast Activation Protein

**DOI:** 10.1158/2767-9764.CRC-24-0248

**Published:** 2024-06-12

**Authors:** Joseph P. Gallant, Hallie M. Hintz, Gihan S. Gunaratne, Matthew T. Breneman, Emma E. Recchia, Jayden L. West, Kendahl L. Ott, Erika Heninger, Abigail E. Jackson, Natalie Y. Luo, Zachary T. Rosenkrans, Reinier Hernandez, Shuang G. Zhao, Joshua M. Lang, Labros Meimetis, David Kosoff, Aaron M. LeBeau

**Affiliations:** 1 Molecular and Cellular Pharmacology Program, University of Wisconsin School of Medicine and Public Health, Madison, Wisconsin.; 2 Department of Pathology and Laboratory Medicine, University of Wisconsin School of Medicine and Public Health, Madison, Wisconsin.; 3 Department of Pharmacology, University of Minnesota School of Medicine, Minneapolis, Minnesota.; 4 Department of Medicine, University of Wisconsin School of Medicine and Public Health, Madison, Wisconsin.; 5 University of Wisconsin Carbone Cancer Center, University of Wisconsin School of Medicine and Public Health, Madison, Wisconsin.; 6 Department of Medical Physics, University of Wisconsin School of Medicine and Public Health, Madison, Wisconsin.; 7 Department of Radiology, University of Wisconsin School of Medicine and Public Health, Madison, Wisconsin.; 8 Department of Human Oncology, University of Wisconsin School of Medicine and Public Health, Madison, Wisconsin.; 9 William S Middleton Memorial Veterans’ Hospital, Madison, Wisconsin.

## Abstract

Cancer-associated fibroblasts (CAF) are a prominent cell type within the tumor microenvironment (TME) where they are known to promote cancer cell growth and survival, angiogenesis, drug resistance, and immunosuppression. The transmembrane prolyl protease fibroblast activation protein (FAP) is expressed on the surface of highly protumorigenic CAFs found in the stroma of nearly every cancer of epithelial origin. The widespread expression of FAP has made it an attractive therapeutic target based on the underlying hypothesis that eliminating protumorigenic CAFs will disrupt the cross-talk between components of TME resulting in cancer cell death and immune infiltration. This hypothesis, however, has never been directly proven. To eliminate FAP-expressing CAFs, we developed an antibody–drug conjugate using our anti-FAP antibody, huB12, coupled to a monomethyl auristatin E (huB12-MMAE) payload. After determining that huB12 was an effective targeting vector, we found that huB12-MMAE potently eliminated FAP-expressing cells as monocultures *in vitro* and significantly prolonged survival *in vivo* using a xenograft engineered to overexpress FAP. We investigated the effects of selectively eliminating CAFs using a layered, open microfluidic cell coculture platform, known as the Stacks. Analysis of mRNA and protein expression found that treatment with huB12-MMAE resulted in the increased secretion of the proinflammatory cytokines IL6 and IL8 by CAFs and an associated increase in expression of proinflammatory genes in cancer cells. We also detected increased secretion of CSF1, a cytokine involved in myeloid recruitment and differentiation. Our findings suggest that the mechanism of FAP-targeted therapies is through effects on the immune microenvironment and antitumor immune response.

**Significance:**

The direct elimination of FAP-expressing CAFs disrupts the cross-talk with cancer cells leading to a proinflammatory response and alterations in the immune microenvironment and antitumor immune response.

## Introduction

Cancer does not develop in isolation, rather it evolves in a complex milieu of reactive stroma and immune cells in the tumor microenvironment (TME). The components of the TME are known to play a range of critical roles in cancer progression and therapeutic resistance (1, 2). Many of these roles are driven by cancer-associated fibroblasts (CAF), a prominent stromal cell population within the TME, which has been found to perform a diverse array of protumor functions (3–6). These varied functions include supporting angiogenesis, driving M2 polarization in macrophages, inhibiting natural killer cell activation, promoting checkpoint inhibitor expression, and directing the recruitment of immunosuppressive myeloid cells (7, 8). CAFs also alter the tumor stiffness through extracellular matrix (ECM) deposition and remodeling which promotes prosurvival and proliferation signaling in cancer cells. The importance of these roles has been demonstrated by a number of studies, which have found that high stromal composition and the presence of a reactive stroma enriched with CAFs is directly correlated with poor prognosis in a range of cancers (9, 10). Given their multifaceted role in driving cancer progression, CAFs have emerged as a potential therapeutic target in the stroma to complement malignant cell–targeted therapies.

Complicating the development of therapies for CAFs is that they exist as heterogenous populations that can be either protumorigenic or antitumorigenic (11). Of the different subsets, CAFs expressing fibroblast activation protein (FAP), a type II transmembrane serine protease, are highly protumorigenic suggesting that their direct elimination represents a strategy that will enhance immune infiltration (12, 13). Demonstrating unique endopeptidase and exopeptidase activity cleaving after proline residues, FAP processes ECM proteins to promote tissue remodeling as well as activate growth factors and cytokines including TGFβ which in turn promotes fibroblast activation and immunosuppression (14, 15). FAP expression in the TME is a hallmark of nearly every solid tumor thus making FAP a potential “pan-cancer” target (16). FAP expression is observed on activated fibroblasts during wound healing, embryogenesis, and fibrosis; however, normal adult tissues have no detectable FAP expression (17). Interestingly, FAP knockout mice develop to maturity with no deleterious effects, which suggests that other compensatory proteases exist for the native function of FAP and that elimination of FAP-expressing cells by a targeted therapy should not affect normal homeostasis (18, 19).

The ubiquitous expression of FAP in solid tumors has made it an attractive target for drug development. Several FAP-targeted therapies ranging from small-molecule radioligands to chimeric antigen receptor T-cell therapies are in various stages of preclinical and clinical development (20–26). The rationale behind targeting FAP for therapy is based on the premise that eliminating CAFs will release the TME from the yoke of CAF-mediated immunosuppression and disrupt cellular crosstalk leading to the starvation of cancer cells (13, 17). Thus far, this mechanism has not been characterized *in vitro* or *in vivo*. To interrogate the effects of selectively eliminating FAP-expressing CAFs, we developed an antibody–drug conjugate (ADC) using a humanized version of our antibody B12 (huB12) coupled to a commonly used ADC linker and payload. Using traditional two-dimensional (2D) cell culture as well as a microfluidic cell culture system, we were able to demonstrate that our FAP-ADC potently and selectively eliminated cells expressing FAP. Through a detailed investigation of mRNA and protein expression of our models, we found that FAP-ADC treatment resulted in increased secretion of proinflammatory cytokines by CAFs with an associated increase in expression of proinflammatory genes in cancer cells. Our data suggest that the mechanism of FAP-targeted therapies may be mediated through effects on the immune microenvironment and antitumor immune response.

## Materials and Methods

### Cell Lines

CWR22Rv1, PC3, SK-MEL-187, and MDA-MB-436 cell lines were purchased from ATCC and were maintained according to ATCC guidelines. hPrCSC-44 cells were obtained from Dr. W. Nathaniel Brennen (Johns Hopkins School of Medicine, Baltimore, MD) and were maintained in RoosterNourish-MSC media (RoosterBio). CWR-R1-luciferase cells were obtained from Dr. Scott Dehm (University of Minnesota, Minneapolis, MN). CWR-R1 cells were lentivirally transduced to express FAP as described previously (27). FAP-expressing cells were continuously supplemented with 3 µg/mL puromycin to ensure stable levels of FAP expression. All cell experiments were performed within 4 months of thawing cell lines from frozen cell stocks. Cell lines were authenticated using short tandem repeat analysis and routinely monitored for *Mycoplasma* contamination prior to our studies.

### Antibody Internalization Studies

Antibodies were fluorescently labeled using succinimidyl esters of Alexa Fluor 647 (Thermo Fisher Scientific #A20006) or pHrodo Red (P36600) prior to analysis by confocal microscopy or Incucyte imaging, respectively. For confocal microscopy studies, hPrCSC-44 and PC3 cells were seeded onto glass-bottom 35 mm dishes (MatTek, P35GCOL-1.5-14-C) at a density of 15,000 cells per dish, 48 hours prior to imaging. On the day of analysis, cells were coincubated with Alexa Fluor 647-labeled antibody (50 nmol/L) and fluorescein-labeled dextran (50 µg/mL; Thermo Fisher Scientific #D1821) for 1 hour at 37°C. Cells were then washed three times in PBS and fixed in 4% paraformaldehyde/PBS for 10 minutes. After fixation, cells were stained for 20 minutes with CellBrite Orange (Biotium #30022) and 2 µmol/L Hoescht 33342 (Thermo Fisher Scientific H3570), in accordance with the vendor recommendations. Samples were mounted on a Nikon Eclipse Ti2 inverted microscope equipped with a Yokagawa W1 CSU spinning disk, cells were imaged with a Plan-Apochromat 100x/1.45 oil objective, fluorescence was recorded using a Hamamatsu ORCA-Quest qCMOS camera. Mander's colocalization coefficient (MCC) was assessed using the JACoP v2.0 plugin in ImageJ. For Incucyte experiments, CWR-R1 and CWR-R1^FAP^ cells were suspended in growth medium supplemented with 25 mmol/L HEPES buffer (pH 7.4) and seeded into optical grade 96-well plates (Thermo Fisher Scientific #165305) at a density of 25,000 cells per well. The following day, varying concentrations of pHrodoRed labeled antibodies were added to cells. pHrodoRed fluorescence was monitored over 3 days in an Incucyte SX5 (Sartorius) using a 20x phase contrast objective and an orange fluorescence optical module (λ_ex_ 557 ± 11nm, λ_em_ 607.5 ± 31.5nm). Data were analyzed by using the Incucyte SX5 Adherent Cell-by-Cell analysis module to measure total integrated orange fluorescence intensity in each experimental condition, values were plotted in OriginLab 2023b for curve fitting.

### Animal Models

All animal studies were approved by the University of Wisconsin Institutional Animal Care and Use Committee. All animal studies were performed in 3 to 4 weeks old Athymic Nude-Foxn1nu mice (Envigo). For subcutaneous tumor implantation, cells suspended in a 1:1 mixture of PBS and Matrigel (Corning) were injected into the rear flank of the mice using a 26-gauge needle. For the engineered cell line model, animals (*n* = 3/group) received subcutaneous injections of CWR-R1^FAP^ cells (1 × 10^6^ cells in 100 µL). For the CAF model, animals (*n* = 3/group) received subcutaneous injections of a 2:1 mixture of hPrCSC-44 and 22Rv1 cells (2 × 10^6^ hPrCSC-44 cells and 1 × 10^6^ 22Rv1 cells in 100 µL). Tumor volumes were measured twice weekly with calipers and tumors were allowed to grow to a size of 100–300 mm^3^ before nuclear imaging experiments.

### Bioconjugation and Radiochemistry

For nuclear imaging studies, the huB12, F19, J591, and IC were conjugated to p-SCN-Bn-Deferoxamine (DFO, Macrocyclic) as described previously (28). Zirconium-89 (^89^Zr) was purchased from the University of Wisconsin Medical Physics Department (Madison, WI). [^89^Zr]Zr-oxalate in 1.0 mol/L oxalic acid (300 µL) was adjusted to pH 7.5 with 1.0 mol/L Na_2_CO_3_. For radiolabeling, huB12-DFO, IC-DFO, F19-DFO or J591-DFO in 0.5 mol/L HEPES (pH 7.5) were added to the pH 7.5 [^89^Zr]Zr-oxalate solution (100 µg per 37 MBq) and incubated at room temperature with rotation for 1 hour. The labeled product was purified using a size-exclusion PD-10 column preequilibrated with PBS buffer.

### PET Image Acquisition

PET imaging experiments were conducted on an Inveon µPET/CT Scanner (Siemens Medical Solutions). Mice (*n* = 3) were administered [^89^Zr]Zr-B12 IgG, [^89^Zr]Zr-F19 IgG, [^89^Zr]Zr-J591 IgG, or [^89^Zr]Zr-IC (4.3–5.5 MBq in ∼100 µL of PBS) via tail vein injection. Mice were anesthetized by inhalation of 2% isoflurane and PET images were recorded serially beginning at 24 hours and continuing until 144 hours postinjection. PET list-mode data were acquired for 30 minutes using a gamma ray energy window of 350–650 keV and a coincidence timing window of 3.438 ns. CT acquisition was performed for 5 minutes at 80 kVp, 500 µA, 384 ms per step, and 340 steps covering 220 degrees. CT images were reconstructed using a Hu scaled Feldkamp algorithm resulting in 192 × 192 matrix and PET utilized Ordered Subset Expectation Maximization (OSEM-3D) with 18 subsets and two iterations resulting in a 128 × 128 matrix. 2D images were prepared in Inveon Research Workplace and quantified using AMIDE. Voxel count rates were converted to activity concentrations and the resulting image data were normalized to the administered activity to parameterize images in terms of %ID/cc. Drawn ellipsoid region of interests (ROI; *n* = 3 per timepoint) were used to determine the mean %ID/cc in various tumors. Three-dimensional (3D) reconstructions were generated using AMIRA.

### Competitive Binding Assay

The competition binding assay was performed using radiolabeled [^89^Zr]Zr-huB12 IgG and either HPrCSC-44 of 22Rv1 as follows. Cells (1–1.5 × 10^5^) were added to 96-well plates containing 0.22 µm filters (Corning). Equivalent amounts of [^89^Zr]Zr-huB12 IgG (1 µCi) were added to each cell-containing well and then cold huB12 IgG was added over a concentration range. After 3 hours of incubation, the plates were filtered using cold 0.1% BSA in PBS three times. The filters were then collected and run on a Wizard2 Gamma counter (Perkin Elmer). Results were normalized to cells incubated with no cold compound (defined as 100% bound).

### Cell Titer Blue Assay

The ADCs used in this study were made and characterized by NJ Bio, Inc. Purification and characterization data are available on request. Cells were seeded in 96-well plates at a density of 5–8 × 10^3^ cells/well in 90 µL of complete culture media and incubated at 37°C and 5% CO_2_ overnight. Free drug or ADC 10x stock concentrations were serial diluted in complete culture media and 10 µL was added to triplicate or duplicate wells for appropriate concentration. Plates were incubated for 72 hours. Cell Titer Blue assay was done following the manufacturer's directions (G8080, Promega). Fluorescence was measured using an Infinite Series plate reader (Tecan). IC_50_ values were calculated using GraphPad software (Prism).

### Stacks Platform

Stacks were sourced from Protolabs (1121-5161-007). Prior to use, the Stacks plates were prepared by sonication in 100% isopropanol for 60 minutes and washed in deionized water. All plates, 3D holders, NuncOmnitrays (Thermo Fisher Scientific), and 245 mm square non–tissue culture–treated BioAssay dish (Corning Inc.) were sterilized by exposure to UV light for 20 minutes on each side in a biosafety cabinet.

### Cell Culture and ADC Treatment

22Rv1 and hPrCSC-44 cells were cultured within Stacks microdevices as set forth by the Stacks platform's established protocols and detailed below, while simultaneously being maintained within standard macro T75 flasks (Corning #430639) for continuous growth following supplier guidelines. 22Rv1, acquired from ATCC, were cultured in RPMI1640 with l-Glutamine (Corning #10-040-CV), 10% FBS (Gibco #10438026), and 2% penicillin/streptomycin (HyClone #SV30010). hPrCSC-44 were cultured in RoosterBasal-MSC-CC (RoosterBio #SU-022), 2% RoosterBooster-MSC-CC (RoosterBio #SU-019), 1% Antibiotic/Antimycotic (Gibco #15240062), and 1% GlutaMax (Gibco #35050061). To prevent evaporation of media during culture in Stacks, adequate humidity was maintained through a multipronged approach. Sterile wet towels were placed in each NuncOmnitray, which was situated within a larger BioAssay dish (Corning #431111) filled with sterile water, and maintained in 37°C humid incubators.

Prior to cell seeding, microwells in each Stacks device were filled with a collagen I (Advanced Biomatrix #5005) fibronectin (Sigma-Aldrich #F1141) biomatrix. Following 4–6 hours biomatrix polymerization, each well received media to both the top and bottom side of the well to maintain the matrix integrity. On day 1, the 22Rv1 and hPrCSC-44 cells were each transferred from macro flasks to the top of the collagen matrix of separate microdevice plates. 22Rv1 seeded at a concentration of 300,000 cells/mL in RPMI media. hPrCSC-44 seeded at a concentration of 150,000 cells/mL in Rooster media. Cells were allowed to grow on their distinct plates for approximately 72 hours. Day 4, plates were stacked together and old media was exchanged for new Rooster media for all stacks. Four cellular conditions were implemented: 22Rv1 monocultured, hPrCSC-44 monocultured, 22Rv1 cocultured with hPrCSC-44, and hPrCSC-44 cocultured with 22Rv1. Cell were cultured for an additional 24 hours following stacking before treatment was administered. Day 5, huB12-MMAE, huB12 antibody control, and PBS control treatments were applied to their respective wells at indicated doses. Each treatment was applied to all monoculture and coculture conditions at indicated doses in triplicate. Day 8, plates were separated for three endpoint analyses: cytotoxicity/viability assay, mRNA analysis, and retained media cytokine assay. Experiment was repeated a total of five times.

### Viability Assay

Following separation of plates, microwells were washed with 1x PBS and then stained with Calcein-AM (Invitrogen #C3099) for 1 hour. Using collagenase from *Clostridium histolyticum* (Sigma-Aldrich #C9697-50MG) to first digest the biomatrix, the stained cells were then extracted to a 384-well low-volume microplate, with each microwell being transferred to its own unique microplate well. Calcein-AM fluorescent intensity was captured for each well (including Blank wells) with a CLARIOstar Plate Reader (BMG Labtech) in the UW-Madison Small Molecule Screening Facility. Relative viability was calculated from Calcein readings as follows:



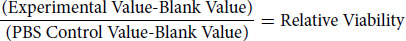



### mRNA Analysis

Upon separating plates, biomatrix in microwells was digested using collagenase from *Clostridium histolyticum* (Sigma-Aldrich #C9697-50MG) and cells were subsequently lysed within microwells using lysis buffer from extraction kit (Thermo Fisher Scientific #61012). Microwell contents were transferred in their entirety to a 96-well plate where released mRNA was captured with Invitrogen DynaBeads mRNA DIRECT kit (Thermo Fisher Scientific #61012). mRNA samples, with associated beads still intact, were then transferred to PCR tubes and reverse transcribed using SuperScript IV VILO Master Mix (Invitrogen #11756050). If necessary, reverse transcribed reactions were promptly flash frozen and stored at −80°C until analysis. Following preamplification with TaqMan PreAmp kit (Applied Biosystems #4488593) for 10 cycles, cDNA samples were mixed with TaqMan Fast Advanced Master Mix (Applied Biosystems #4444557), nuclease-free water, and TaqMan Gene Expression Assay *AR* (Hs00171172_m1), *BCL2* (Hs00608023_m1), *BCL2L1* (Hs00236329_m1), *CCL2* (Hs01574247_m1), *CCL5* (HS00982282_m1), *CCL22* (Hs01574247_m1), *CCND1* (Hs00765553_m1), *CCND2* (Hs00153380_m1), *CSF1* (HS00174164_m1), *CXCL8* (Hs00174103_m1), *CXCL9* (HS00171065_m1), *CXCL10* (Hs01124252_g1), *CXCL11* (Hs04187682_g1), *CXCL12* (Hs03676656_mH), *IL6* (Hs00174131_m1), *IL10* (Hs00961622_m1), *KLK3* (Hs02576345_m1), *PDL1/CD274*, *TGFB1* (HS00998133_m1), *TNF* (Hs00174128_m1), *VEGFA* (HS00900055_m1; Life Technologies). Using QuantStudio 5 Real-Time PCR system via the Comparative CT Fast protocol available on the machine, reactions were run for 40 cycles. Analysis was performed using the Thermo Fisher Connect online application.

### Luminex Assay

Following treatment, 20 µL of media was collected from each condition and diluted with 40 µL of Rooster media (to obtain sufficient volume) and centrifuged at 300 × *g* for 3 minutes. Diluted media was snap frozen on dry ice and stored at −80°C until analysis. Using a custom multianalyte Luminex Assay kit (R&D Systems #LXASHM-12), samples, along with standards and blanks, were run in duplicate and analyzed in accordance with manufacturer's protocol. The following analytes were evaluated: CCL2/JE/MCP-1, CCL22/MDC, CXCL9/MIG, CXCL10/IP-10/CRG-2, CXCL11/I-TAC, EGF, IL6, IL8/CXCL8, MCSF, MMP-9, TNFα, VEGF. After subtracting out blank average values, standard curves were generated with the 5-PL curve fit analysis tool in Prism (GraphPad Software) and sample cytokine concentrations were quantified.

### Statistical Analyses

All assays were run over five separate experiments. Statistical analysis for viability assay was run with Student’s *t*-test between control and ADC conditions. qPCR assay and Luminex assay statistical analyses were each performed as one-way ANOVA repeated measures within cellular condition, using Prism (GraphPad Software).

### Data Availability

The data generated in this study are available within the article and its Supplementary Data files.

## Results

### HuB12 Internalization Studies

Previously, we identified an antibody, B12, from a mouse antibody phage display library that was found to be cross-reactive with human and mouse FAP (27). FAP is a member of the prolyl protease family, which contains dipeptidyl peptidase IV (DPPIV), DPP7, DPP8, and DPP9 among several others (29). Many members have high homology—FAP and DPPIV share 70% amino acid sequence homology—and many are expressed under normal physiologic conditions. The high homology between FAP and DPPIV has made developing FAP-specific antibodies difficult using traditional hybridoma technology (30). We found that B12 did not bind recombinant DPPIV or DPPIV expressed on surface of the prostate cancer cell line PC3 (27). For clinical translation, B12 was fully humanized using human sequences as acceptor frameworks for the variable domains. The acceptor sequences were from a human IgG1 to ensure that the humanized sequences were nonimmunogenic and retained the structure of the complementarity determining region loops (Supplementary Fig. S1).

**FIGURE 1:**
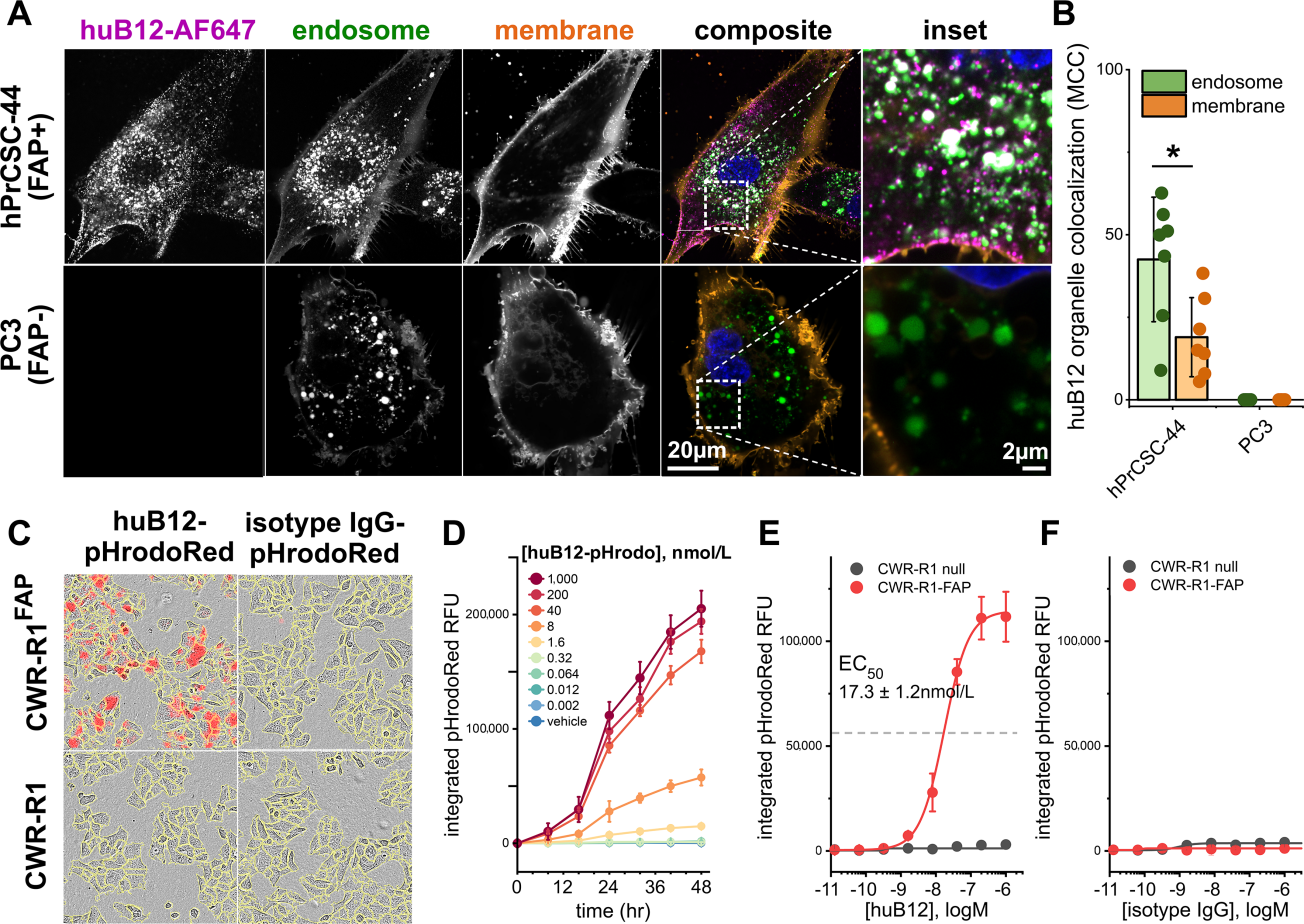
HuB12 internalizes into FAP-expressing cells. **A,** Confocal microscopy images of hPrCSC-44 cells (top) or PC3 cells (bottom) after incubation with Alexa Fluor-647 (AF647)-labeled huB12 (50 nmol/L) for 1 hour. Single-channel images of huB12-AF647 localization, fluorescein-dextran labeled endosomes, and CellBrite Orange-labeled plasma membrane are shown. Composite images depicting whole-cell and enlarged regions of interest are shown as colored fluorescence overlays. **B,** Quantification of the organellar distribution of huB12-AF647, assessed using MCC with either fluorescein dextran (endosome marker) or CellBrite Orange (membrane marker). Values represent average ± SD of *n* ≥ 6 images, *, *P* <0.05 using two-tailed Student’s *t*-test. **C,** Representative images of CWR-R1^FAP^ or parental CWR-R1 cells after 16 hours incubation with the indicated antibody (40 nmol/L). A panel of phase-contrast images overlaid with an outline of individual cells (yellow) and pHrodoRed fluorescence (red) is shown. **D,** Time course of pHrodoRed fluorescence intensity in CWR-R1^FAP^ cells incubated with the indicated concentration of huB12-pHrodoRed, data shown from a representative experiment. Dose–response curves of peak pHrodoRed fluorescence values in CWR-R1 (gray) or CWR-R1^FAP^ (red) cells after 24 hours incubation with increasing concentrations of huB12-pHrodoRed (**E**) or isotyped control IgG-pHrodoRed (**F**). Values represent average ± SEM from *n* = 3 replicates.

Because our goal was to make huB12 into an ADC, we thoroughly investigated its mechanism of internalization to ensure that it would effectively deposit a payload within the cell. Confocal microscopy studies were performed using the FAP-expressing human prostate CAF cell line, hPrCSC-44, and the FAP-null prostate cancer cell line PC3. Cells were coincubated with Alexa Fluor 647-labeled huB12 (huB12-AF647, 50 nmol/L) and fluorescein-dextran, as a marker of fluid-phase endocytic cargo, prior to fixation and organellar staining. After a 1-hour incubation with huB12-AF647, hrPrCSC-44 displayed intense fluorescent puncta throughout the cell (Fig. 1A). Visual inspection of multichannel composite images suggests huB12-AF647 localized to both the plasma membrane and intracellular vesicular structures. In contrast, no significant huB12-AF647 fluorescence was detected in PC3 cells, despite being viable and endocytosis-competent, as indicated by endocytic internalization of fluorescein-dextran. Colocalization of huB12-AF647 with endosomal compartments or with the cell membrane was quantified using MCC. In hPrCSC-44 cells, MCC analysis revealed significantly higher pixel-to-pixel colocalization between huB12-AF647 and fluorescein-dextran compared with colocalization of huB12-AF647 and carbocyanine-stained plasma membranes (Fig. 1B).

As an orthogonal method of measuring huB12 internalization, we performed high content live-cell imaging experiments using antibodies labeled with the pH-sensitive fluorophore, pHrodoRed. This approach uses pHrodoRed fluorescence as an indicator of antibody translocation through acidic endosomal compartments. huB12-pHrodoRed internalization was assessed in CWR-R1^FAP^ cells, an engineered prostate cancer cell line with stable heterologous FAP expression, as well as in parental FAP-null CWR-R1 cells. huB12-pHrodoRed internalized into CWR-R1^FAP^ cells, and this effect was dependent upon FAP expression, as evidenced by the lack of pHrodoRed fluorescence in CWR-R1 cells (Fig. 1C). pHrodoRed fluorescence was readily detectable within 8 hours and continued to increase as a function of time (Fig. 1D). Importantly, huB12-pHrodoRed internalization displayed a dose-dependent and saturable signal, with an EC_50_ of 17.3 ± 1.2 nmol/L (Fig. 1E). Incubation of these same cell lines with pHrodoRed-labeled isotyped control IgG failed to produce a fluorescent signal, further suggesting that internalization of huB12-pHrodoRed is due to specific engagement of FAP, and not due to bulk endocytic uptake of extracellular cargo (Fig. 1C and F). These data demonstrate FAP-dependent internalization of huB12 into prostate cancer cells, enabling further development of huB12 as an ADC.

### PET Imaging of FAP in a Prostate Cancer Model

**FIGURE 2:**
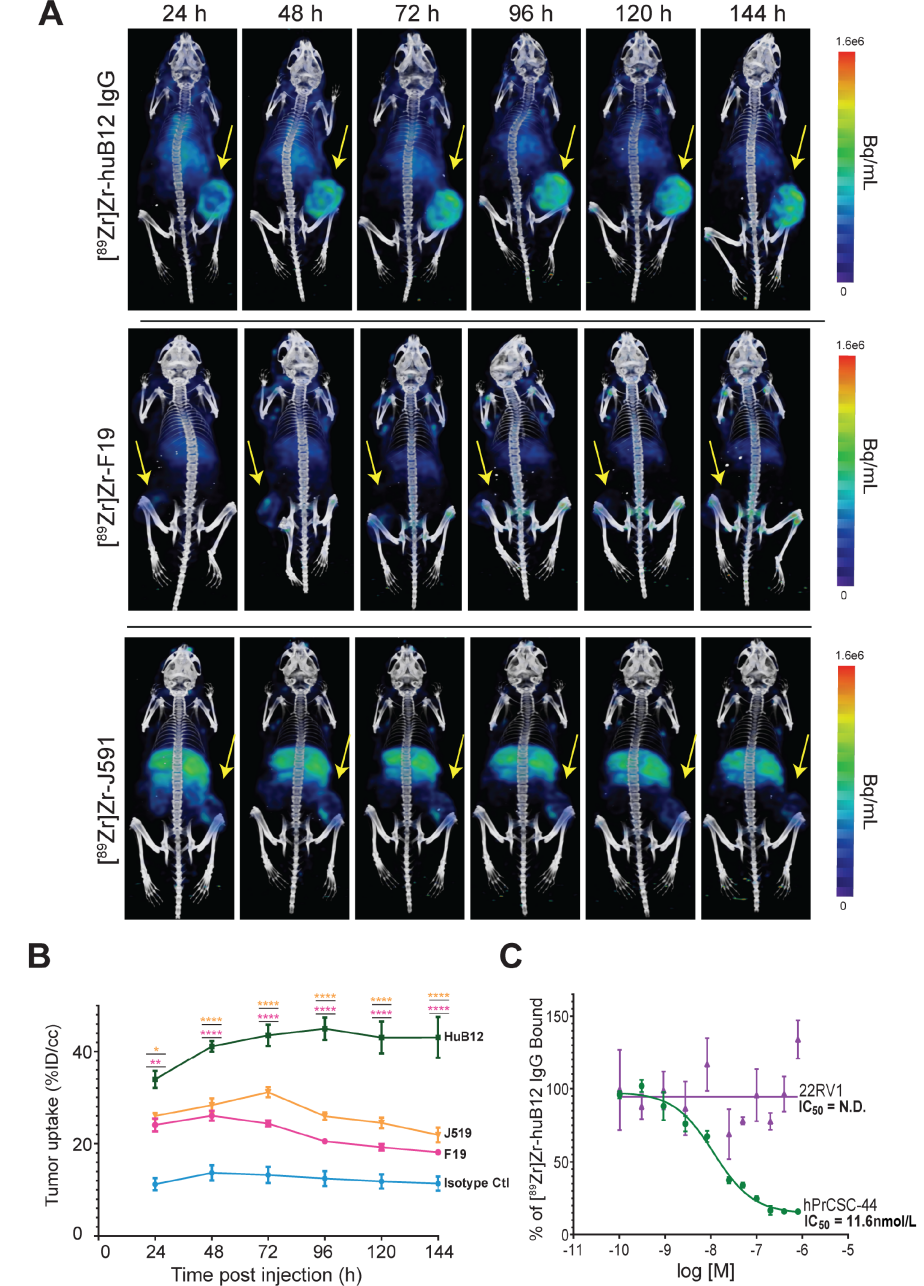
PET/CT imaging of FAP *in vivo*. **A,** Representative PET/CT images of mice bearing subcutaneous 22Rv1/hPrCSC-44 xenografts (white arrowhead). Mice (*n* = 3/group) received 3.5 MBq (25–50 µg, 7.7 µg/MBq) of [^89^Zr]Zr-HuB12, 3.6 MBq (25–50 µg, 7.5 µg/MBq) [^89^Zr]Zr-F19 or 3.4 MBq (25–50 µg, 7.9 µg/MBq) [^89^Zr]Zr-J591 via tail vein and were imaged at the indicated timepoints. **B,** Quantitative analysis of subcutaneous xenografts from mice (*n* = 3/group) revealed significantly higher [^89^Zr] uptake in the tumors of [^89^Zr]Zr-HuB12 administered animals compared with the [^89^Zr]Zr-F19 or [^89^Zr]Zr-J591 at all timepoints postinjection. Values represent mean ± SEM. *, *P* ≤ 0.05; **, *P* ≤ 0.01; ****, *P* ≤ 0.0001. **C,** Radio-competition cell binding results using [^89^Zr]Zr-HuB12 and either the FAP-positive hPrCSC-44 or FAP-negative 22Rv1 cell line. The IC_50_ was only determinable for hPrCSC-44 and was measured at 11.6 nmol/L.

Next, we performed a comparative PET imaging study with [^89^Zr]Zr-huB12 and two other antibodies that recognize cell surface antigens using a xenograft model that mimicked the TME (Fig. 2A). Mice were implanted with xenografts composed of hPrCSC-44 cells and the FAP null castration-resistant prostate cancer cell line 22Rv1 (28, 31). 22Rv1 cells possess androgen receptor (AR) splice variants—a common resistance mechanism antiandrogen therapies—and also express prostate-specific membrane antigen (PSMA) which has been targeted extensively for imaging and therapy in prostate cancer using small-molecule ligand and antibody radiotherapies (32, 33). We compared tumor uptake between huB12 and two IgGs that were previously investigated in the clinic: F19, which targets FAP and J591, a PSMA-targeted antibody (34, 35). Mice bearing the hPrCSC-44/22Rv1 mixed xenograft were injected with [^89^Zr]Zr-huB12 IgG, [^89^Zr]Zr-F19, [^89^Zr]Zr-J591 or [^89^Zr]Zr-IC IgG and imaged by PET serially at 24, 48, 72, 96, 120, and 144 hours postinjection. Analysis of the 3D imaging data revealed high uptake in the FAP-positive stroma tumors by 48 hours in the animals administered [^89^Zr]Zr-huB12 IgG. Retention of [^89^Zr]Zr-huB12-IgG in the tumor and clearance from the liver was observed over time improving the signal-to-noise. Antithetically, mice administered [^89^Zr]Zr-F19 or [^89^Zr]Zr-J591 had lower uptake in the hPrCSC-44/22Rv1 tumors with the signal largely clearing by 144 hours. Quantitative ROI analysis of imaging agent uptake in the tumor was significantly higher in the [^89^Zr]Zr-huB12 IgG group compared with all other agents tested (Fig. 2B). Importantly, at the 72 hours timepoint analysis of the % injected dose (%ID/cc) was significantly higher with [^89^Zr]Zr-huB12 IgG than either [^89^Zr]Zr-F19 or [^89^Zr]Zr-J591. The [^89^Zr]Zr-huB12 IgG %ID/cc average was 43.53 ± 4.06 versus 24.35 ± 1.16 (*P* < 0.0001) and 31.13 ± 1.86 (*P* < 0.0001), respectively for [^89^Zr]Zr-F19 and [^89^Zr]Zr-J591.) An *in vitro* cell binding assay with the cell lines used to generate the xenograft model additionally confirms the specificity of [^89^Zr]Zr-huB12 for FAP-expressing PrCSC-44 cells (Fig. 2C).

### *In Vitro* and *In Vivo* Characterization of huB12-MMAE

**FIGURE 3:**
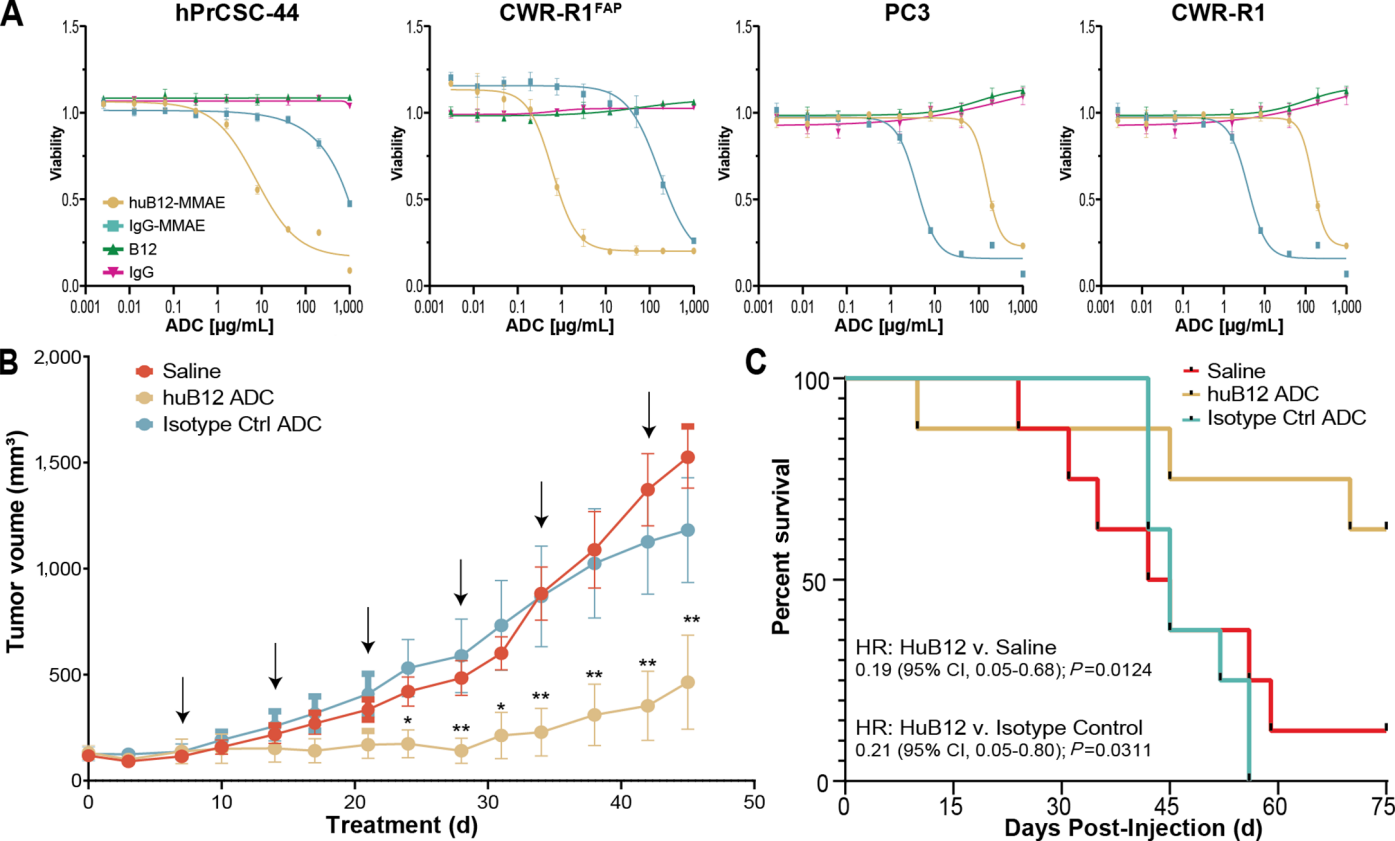
*In vitro* and *in vivo* therapeutic efficacy of huB12-MMAE. **A,** FAP-targeted ADCs show a potent and FAP-selective cytotoxic activity. Cell viability hPrCSC-44, CWR-R1FAP, PC3, and CWR-R1 cell lines. Cells were treated for 72 hours with a serial dilution of either huB12 IgG or an isotype control IgG with or without an MMAE warhead. Data are represented as mean ± SEM (*n* = 3 biological replicates). **B,** HuB12 ADC therapy demonstrates significant tumor control compared with the saline arm. Black arrows signify treatment administration (*, *P* ≤ 0.05; **, *P* ≤ 0.01, ANOVA mixed methods test). **C,** Kaplan–Meier survival curve for mice treated with therapeutic antibodies, huB12 ADC or Isotype Control ADC compared with saline control mice. Mice treated with huB12 ADC survived significantly longer than the controls, HR analysis used for statistical analysis.

Having validated huB12 as a selective and sensitive PET agent, we moved forward with testing huB12 as a delivery vector for cytotoxins. ADCs were designed using huB12 with a cathepsin cleavable valine-citruline-para-aminobenzyl carbamate (val-cit-PAB) linker attached to three different payloads. For the payloads, the tubulin inhibitors monomethyl auristatin E (MMAE) and MMAF were used in addition to mertansine (DM1). These linker-payload combinations were used because they are common in FDA-approved ADCs—nearly half of the approved ADCs employ val-cit-PAB-MMAE (36). For each ADC, the linker-payloads were attached to huB12 via cysteine residues resulting in a drug-to-antibody ratio (DAR) of 4. The ADCs were evaluated in a prostate cancer cell line engineered to overexpress FAP (CWR-R1^FAP^), our immortalized CAF model (hPrCSC-44), and two endogenous FAP-expressing cell lines, MDA-MB-436 (breast cancer) and SK-MEL-187 (melanoma). Out of the three payloads, huB12-MMAE consistently resulted in low IC_50_ values across the cell lines tested (Supplementary Fig. S2). We next confirmed the specificity of huB12-MMAE using FAP-negative and FAP-positive cell lines and an IgG1 isotype control ADC (IgG-MMAE). Potent cell death was observed in the FAP-positive cells CWR-R1^FAP^ and hPrCSC-44 with IC_50_ values of 0.61 and 0.87 µg/mL respectively. No killing was observed in the parental CWR-R1 cells or the DPPIV-expressing cell line PC3 (Fig. 3A).

It is common in FAP drug development to use cell lines engineered to overexpress FAP due to the paucity of accurate and cost-effective FAP-expressing models. Therefore, we investigated the therapeutic efficacy of huB12-MMAE in a prostate cancer xenograft model engineered to overexpress FAP. Mice bearing engineered CWR-R1^FAP^ xenografts (tumor volumes ∼100–200 mm^3^) were randomly assigned to the three treatment arms; saline, huB12-MMAE, or IgG-MMAE (nontargeted control). Treatment was administered via tail vein injection once weekly for 6 weeks and tumor volumes were measured twice weekly until tumors reached endpoints of either 1,500 mm^3^ or ulcerations. The arm treated with huB12-MMAE (20 mg/kg dose) displayed robust and significant antitumor effect. At day 24 after initial treatment, huB12-MMAE–treated arm had significantly reduced tumor growth compared with the saline arm (*P* < 0.05; Fig. 3B). At the conclusion of the study, the saline group tumor volume average exceeded endpoint criteria at 1,500 mm^3^ whereas the huB12-MMAE group average was significantly lower at 460 mm^3^ (*P* < 0.005). In addition, huB12-MMAE had a profound effect on overall survival, 75% of huB12 subjects survived at the conclusion of the study at 75 days compared with 12.5% for the IgG-MMAE arm and 0.0% for the saline arm (Fig. 3C). Average survival was improved in the huB12-MMAE–treated arm at 62.5 days compared with 45.9 days for IgG-MMAE and 47.5 days for saline. Collectively, these results reveal huB12-MMAE as a potent antitumor therapeutic with evidence for reduced tumor size and improved survival.

### Investigating the Effects of Selectively Eliminating CAFs

**FIGURE 4:**
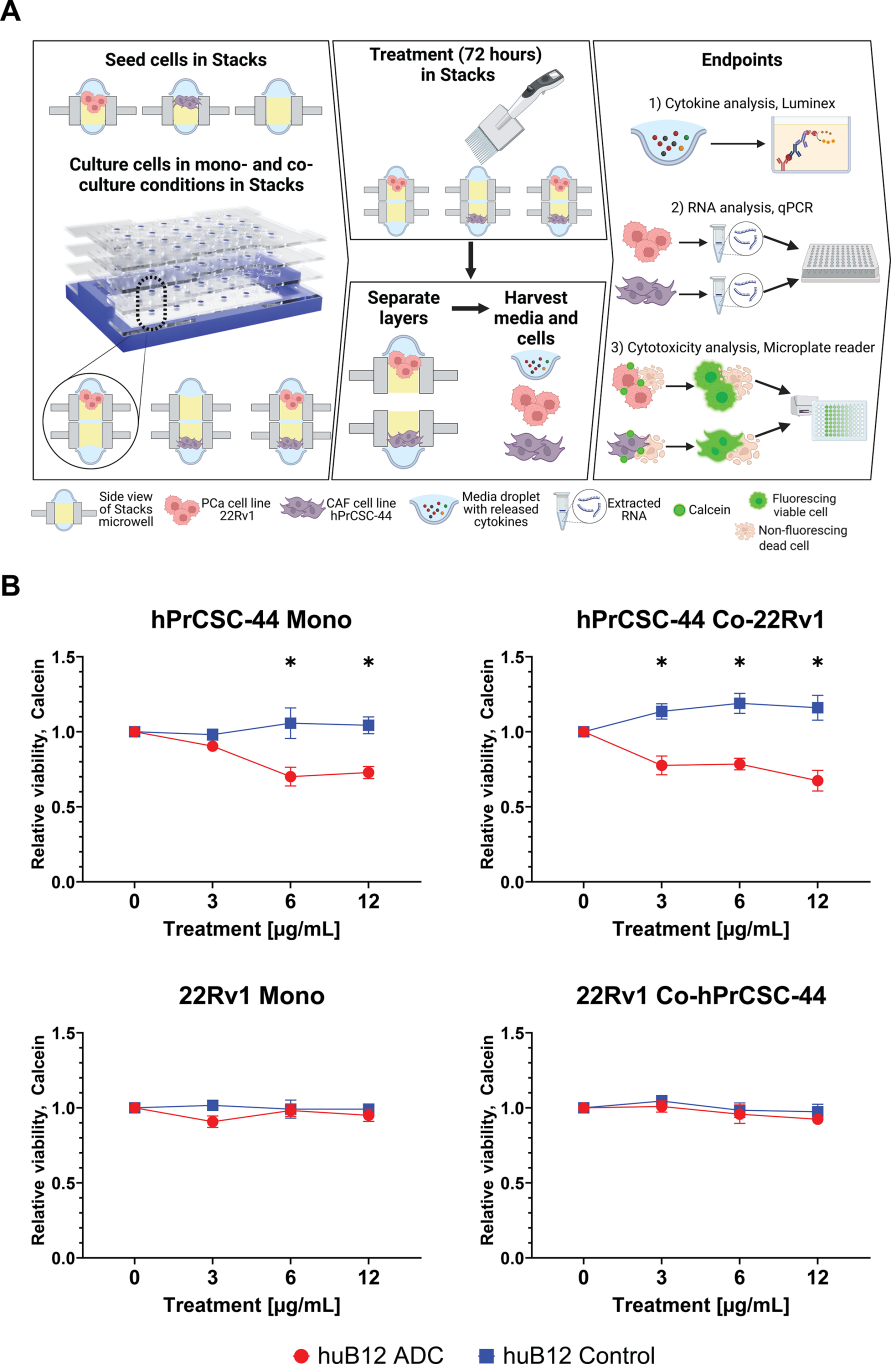
Specificity of huB12-MMAE treatment in the Stacks. **A,** Workflow of treatment and analysis of CAF-tumor models using huB12-MMAE. **B,** Viability analysis of CAFS (hPrCSC-44/top) and tumor cells (22Rv1/bottom) in monoculture (left) and coculture (right) using indicated concentrations of huB12-MMAE for 72 hours.

We next aimed to investigate the potential mechanisms of action of targeting FAP with an ADC. For this work, we leveraged a microfluidic cell culture platform, known as Stacks, which we have previously validated for TME investigation (Fig. 4A; ref. 37). Utilization of the Stacks platform allowed us to perform simultaneous analysis of cell cytotoxicity, mRNA expression, and secretory factors for mechanistic studies (37). We first identified a therapeutic window for a 72-hour treatment with huB12-MMAE in Stacks using an hPrCSC-44 monoculture (Supplementary Fig. S3). On the basis of these data, we selected three concentrations (3, 6, and 12 µg/mL) of huB12-MMAE to evaluate in our monoculture and coculture models of fibroblasts and tumor cells. To perform this analysis, hPrCSC-44 and 22Rv1 cells were first seeded on 3D biomatrix in separate Stack plates and allowed to adhere. Plates were then combined to establish cocultures and corresponding two-layer monocultures (22Rv1 + blank layer and hPrCSC44 + blank layer; Fig. 4A). After 24 hours of culture/coculture, treatment was then applied for 72 hours followed by separation of layers for evaluation of cell viability, mRNA expression, and secreted factor concentrations. Our viability analysis demonstrated that huB12-MMAE treatment produced a clear cytotoxic effect on the hPrCSC-44 cells in both the monoculture and coculture conditions (Fig. 4B). In the hPrCSC-44 cells in monoculture, there was limited viability reduction at the 3 µg/mL dose, but a 20%–30% reduction in viability at 6 and 12 µg/mL doses. In the coculture models, huB12-MMAE treatment produced a 20%–30% reduction in viable hPrCSC-44 cells among all evaluated doses. In contrast to these findings, we did not detect any evidence of huB12-MMAE cytotoxicity on the 22Rv1 cells in either the monoculture or coculture conditions (Fig. 4B). The cytotoxic effect of huB12-MMAE therefore appeared to be specific to FAP-expressing cells and we did not observe a bystander effect in this model at this timepoint.

**FIGURE 5:**
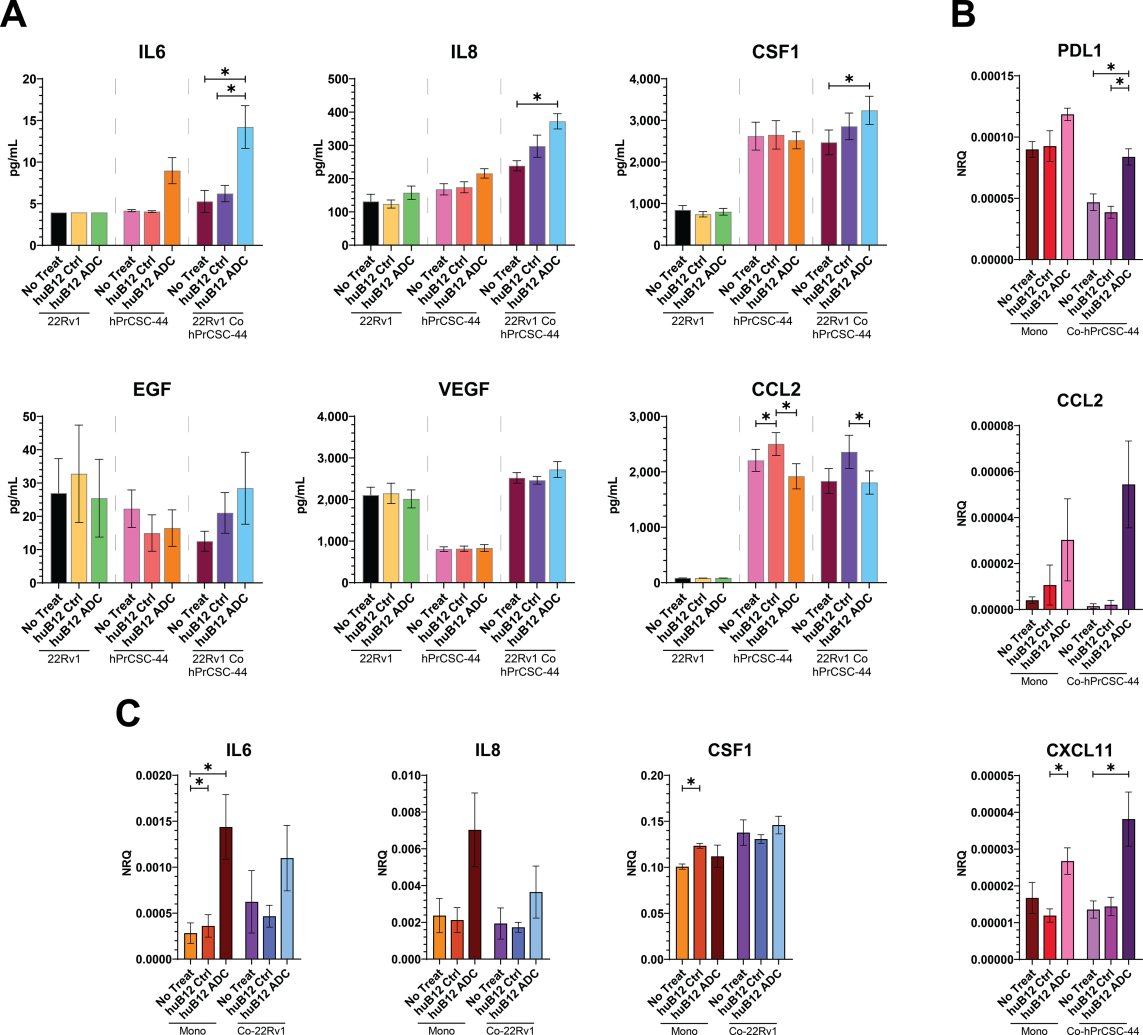
Downstream effects of huB12-MMAE treatment on gene expression in CAF-tumor models in Stacks. **A,** Concentration of selected factors in supernatant in monoculture and coculture conditions. **B,** Relative mRNA expression of select genes in hPrCSC-44 cells in monoculture and coculture treated and untreated conditions. **C,** Relative mRNA expression of select genes in 22Rv1 cells in monoculture and coculture treated and untreated conditions. Data for B and C expressed as normalized relative quantity (NRQ). *, *P* < 0.05.

In our subsequent analysis, we interrogated our models for potential functional consequences of FAP-ADC directed treatment within the TME. On the basis of the established roles of CAFs within the prostate TME, we analyzed the expression of mRNA and secreted factors related to CAF regulation of (i) cell proliferation/survival; (ii) angiogenesis; and (iii) immune modulation (refs. 38–41; Fig. 5). Because the 6 µg/mL dose was the lowest dose associated with the maximum cytotoxic effect in both the monoculture and coculture models, we focused our analysis on this dosage compared with huB12 and PBS controls. While we did not detect any difference in relative cancer cell number in the huB12-MMAE treatment groups to suggest an effect on cancer cell proliferation, the time interval of evaluation may have been too short to fully evaluate for a proliferative effect. We also evaluated the supernatant media for CAF-secreted factors known to play a role in tumor cell proliferation and we analyzed the tumor cells for mRNA markers of proliferation and survival. Through this analysis, we did detect an increase in supernatant levels of IL6 and IL8, which have known roles in tumor cell proliferation and survival (Fig. 5A; refs. 42–44). However, we did not detect any significant change in 22Rv1 mRNA expression of proliferative or survival markers, including AR, PSA, CCND1, CCND2, BCL2, BCLXL in the coculture treated conditions to indicate an increase in proliferation (Supplementary Fig. S4). Furthermore, we did not detect an increase in other CAF-secreted factors in the supernatant, such as EGF, with known roles in tumor proliferation and survival (Fig. 5A; ref. 45). It did not appear that targeting CAFs with huB12-MMAE alone had an appreciable effect on tumor cell proliferation or survival in our 2D Stacks models.

We did not identify any effect of ADC treatment on CAF-mediated angiogenesis. Because IL6 and IL8 can drive angiogenesis through upregulation of VEGFA expression in tumor cells, we evaluated the tumor cells for changes in mRNA expression in VEGFA (46–48). However, we did not observe any change in 22Rv1 expression of VEGFA in the treatment conditions compared with the control (Supplementary Fig. S5). While CAFs can also drive angiogenesis in the TME directly through secretion of VEGFA, we did not identify any change in VEGFA mRNA expression in the hPrCSC-44 cells in coculture (Supplementary Fig. S5). Furthermore, evaluation of the supernatant did not demonstrate any change in VEGFA concentration following huB12-MMAE treatment in any of the conditions (Fig. 5A). In addition to proliferation and angiogenesis, IL6 and IL8 are known to have proinflammatory effects on the surrounding microenvironment (42, 49, 50). We also detected an increase in CSF1, which directs myeloid recruitment/differentiation, in the supernatant of the coculture ADC-treated wells (51). We then investigated the 22Rv1 cells for mRNA expression of genes that are regulated by the inflammatory state of the surrounding TME to evaluate for a proinflammatory effect of ADC treatment. This analysis demonstrated a significant increase in PDL1 and CXCL11 mRNA in the 22Rv1 cells as well as a nonsignificant increase in CCL2 (Fig. 5B). PDL1, which protects tumor cells from T-cell cytotoxicity, and CCL2, which has roles in myeloid cell recruitment and function, are both induced by proinflammatory stimuli such as IL6 (52, 53). Furthermore, CXCL11 has established roles in CD8 T-cell recruitment for tumor cell destruction (54). The increased mRNA expression of these cytokines therefore suggests that huB12-MMAE treatment increases proinflammatory pathways in tumor cells through the increased expression of IL6, IL8, and CSF1 by CAFs.

No increase was detected in the transcription of all evaluated proinflammatory cytokines in the 22Rv1 cells (Supplementary Fig. S6A). We also did not detect increased transcription in any of the evaluated immunosuppressive genes (Supplementary Fig. S6B), further supporting the proinflammatory status of the 22Rv1 cells. CAFs are well-known producers of IL6, IL8 and analysis of CAF mRNA demonstrated upregulation of IL6 and IL8, suggesting that the noted increase in these cytokines was due, at least in part, to increased expression by CAFs following treatment rather than released protein from dead or dying cells (Fig. 5C; ref. 55). There was no identified change in CSF1 transcription in either the 22Rv1 cells or hPrCSC-44 cells and no change in cancer cell IL6 (Supplementary Fig. S6A and S6C). There was also no increased transcription of CXCL12 or TGFB1 in the hPrCSC-44 cells, which are two other genes with well-established roles in CAF-mediated tumor progression (Supplementary Fig. 6C; ref. 45).

## Discussion

It has been estimated that FAP is overexpressed in 90% of all solid tumors (56, 57). The TME consists of heterogenous cell populations and structural proteins that promote tumor growth and metastasis. Within this heterogenous cell population exists a heterogenous population of CAFs. Though a few contradictory reports exist, overwhelming evidence suggests that CAFs expressing FAP are immunosuppressive, promote drug resistance, and help drive tumor growth and metastasis. FAP expression has been demonstrated to be predictive of a negative outcome in several cancer types including lung, liver, colon, and pancreatic ductal adenocarcinoma (58–60). For our studies, we decided to focus on prostate cancer as our model. The role of FAP in prostate cancer carcinogenesis and disease progression is unknown; however, prostate cancer is emblematic of a cancer that could benefit from FAP-ADC therapy. Prostate cancer is a highly heterogeneous disease consisting of malignant cells that overexpress AR or possess AR splice variants (61). Since the advent of second-generation antiandrogen therapy, increased cases of prostate cancer differentiating into aggressive disease lacking AR expression with neuroendocrine markers (NEPC) have been observed (62). NEPC lacks the expression of PSMA, and no targeted therapies exist for it (63). Previously, we found that FAP is overexpressed by IHC in the TME of metastatic prostate cancer regardless of location and treatment modality (28). By analyzing gene expression data, we also found that FAP was expressed in all subtypes of prostate cancer regardless of AR status or neuroendocrine differentiation. A recent study found that FAP expression correlated with worse overall survival in patients with metastatic NEPC (64). If the rationale for targeting FAP is accurate, a FAP-ADC has the potential to overcome tumor heterogeneity by eliminating AR-driven and non–AR-driven disease simultaneously by targeting a population of CAFs that aid and abet heterogeneous disease. It is possible that the direct elimination of FAP-expressing CAFs could lead to the emergence of dominant FAP-negative CAF populations. Because FAP-expressing CAFs are known to be among the most tumorigenic and immunosuppressive, it is likely that this enrichment of FAP-negative CAFs would occur at a later stage when the TME has warmed enough immunologically for other therapies to be effective.

Considering the presence of CAFs within a diverse array of tumors, and the highly restrictive expression of FAP within tumor versus normal tissues, FAP represents a promising therapeutic target for cancer treatment. Although FAP has been known for three decades, no targeted therapies for FAP have been approved by the FDA (65). Early efforts using the small-molecule boronic acid Talabostat to inhibit the enzymatic activity of FAP for therapeutic benefit failed in the clinic (66). More recently small-molecule and peptide inhibitors of FAP have been developed and are undergoing clinical evaluation primarily as radioligand therapies (21, 67). The low delivered dose and rapid efflux from the tumor suggests that these molecules may be suboptimal targeting agents for radionuclide delivery. The antibody sibrotuzumab (F19) was evaluated in phase I/II clinical trials for antitumor efficacy in patients with metastatic colon and non–small cell lung cancer. No therapeutic efficacy was observed and sibrotuzumab was withdrawn from clinical trials (68). One explanation for the failure of sibrotuzumab is the lack of internalization upon antigen engagement. This characteristic limited the utility of sibrotuzumab as a radiotherapy and an ADC. Though a number of FAP-targeted therapies have been developed, it is not hyperbolic to state that optimal targeting vectors have not been identified and significant room for improvement exists.

One of the primary challenges in the development of FAP-targeted therapies has been the lack of translational models that support investigating therapeutic efficacy as well as the elucidation of the mechanism of action. Only a handful of immortalized FAP-positive fibroblast cell lines and cancer cell lines that express endogenous FAP exist. To our knowledge, hPrCSC-44 cells are the only known immortalized FAP-expressing cell line of prostate origin. For *in vivo* studies, few syngeneic models exist and there has never been a report characterizing FAP in a genetically engineered mouse model. Nearly all of the studies for FAP-targeted therapies have been performed using cell lines engineered to overexpress FAP (20, 67, 69). Though they do not recapitulate CAFs in the TME, these models are widely used for imaging and drug development. Patient-derived xenografts do express FAP in the stroma; however, the amount of stroma is generally significantly less than in clinical disease (70). To overcome these limitations and to investigate the molecular cross-talk between CAFs and cancer cells, we employed novel microfluidic cell culture technology in combination with a FAP-expressing CAF line to evaluate the efficacy and mechanism of action of our ADC huB12-MMAE. Through this investigation, we were able to demonstrate that huB12 was an excellent targeting vector because of its cellular internalization upon antigen engagement and high tumor uptake by PET. On the basis of these favorable properties, huB12-MMAE was effective at eliminating FAP-expressing cells *in vitro* and *in vivo* with high specificity. A frequent dosing regimen of huB12-MMAE was required to achieve a therapeutic effect *in vivo*. However, this can be overcome by increasing the DAR from 4 to 6 or 8 as is common. Furthermore, payloads with greater cytotoxic profiles can be used such as the anthracycline topoisomerase inhibitor PNU-159682 or the DNA interstrand cross-linking payload pyrrolobenzodiazepine (71, 72).

We were able to demonstrate that targeting CAFs with huB12-MMAE resulted in increased levels of secreted IL6, IL8, and CSF1 within the supernatant media. While these cytokines can mediate an array of processes within the TME, including angiogenesis and tumor proliferation, our analysis of the tumor cells suggests that the primary effect of this cytokine increase within the TME is the promotion of a proinflammatory microenvironment. On the basis of these findings, it appears that the mechanism of action of FAP-directed therapies is the modulation of the immune microenvironment and the promotion of proinflammatory tumor-directed responses. These findings are further supported by current literature documenting the prominent role that CAFs play in modulating tumor immunity as well as the efficacy of immunotherapies (8). If true, this mechanism of action would support rational therapeutic combinations of CAF-directed therapies with immune-targeted therapies in prostate cancer as well as other cancers. However, further investigation of huB12-MMAE within models that incorporate additional immune cell populations, included tumor-associated macrophages and T cells (among others) will be needed to confirm an immune-mediated mechanism of action and potential role for immunotherapy combinations. In our studies, we also grew the cells as 2D monolayers in the Stacks microwells. In the future, we will investigate the use of 3D spheroid models that incorporate CAFs and cancer cells to recapitulate a 3D tumor. Studies have shown that paracrine factors can drive greater invasiveness in 3D models compared with 2D (73). Thus, it is possible that cancer cell spheroids cocultured with CAFs in 3D maybe become addicted to CAF signaling and our FAP ADC may have a greater therapeutic effect on cancer cells even in the absence of immune cells.

To our knowledge, this is the first study that was able to evaluate FAP ADC efficacy, specificity, and mechanism of action within a multicellular tumor model. These studies were supported by the utilization of microfluidic technologies that enabled high throughput and multiplexed analysis of cell-specific viability, mRNA expression, and secreted factor profile. Furthermore, due to the low input requirements, low quantities of drug were required for each replicate, thereby enabling simultaneous analysis of numerous treatment conditions. These microfluidic models therefore represent a promising tool for future drug development.

## Supplementary Material

Supplementary Figure 1 Antibody Humanization

Supplementary Figure 2 ADC efficacy in multiple cell lines

Supplementary Figure 3 Determining the therapeutic window in the Stacks

Supplementary Figure 4 Proliferation and survival marker gene expression

Supplementary Figure 5 VEGFA expression

Supplementary Figure 6 mRNA expression of cytokines

## References

[1] Tiwari A , Trivedi R, Lin SY. Tumor microenvironment: barrier or opportunity towards effective cancer therapy. J Biomed Sci 2022;29:83. 36253762 10.1186/s12929-022-00866-3 PMC9575280

[2] Taylor RA , Risbridger GP. Prostatic tumor stroma: a key player in cancer progression. Curr Cancer Drug Targets 2008;8:490–7. 18781895 10.2174/156800908785699351

[3] Sahai E , Astsaturov I, Cukierman E, DeNardo DG, Egeblad M, Evans RM, . A framework for advancing our understanding of cancer-associated fibroblasts. Nat Rev Cancer 2020;20:174–86. 31980749 10.1038/s41568-019-0238-1 PMC7046529

[4] Escamilla J , Schokrpur S, Liu C, Priceman SJ, Moughon D, Jiang Z, . CSF1 receptor targeting in prostate cancer reverses macrophage-mediated resistance to androgen blockade therapy. Cancer Res 2015;75:950–62. 25736687 10.1158/0008-5472.CAN-14-0992 PMC4359956

[5] Senger G , Ragoussis J, Trowsdale J, Sheer D. Fine mapping of the human MHC class II region within chromosome band 6p21 and evaluation of probe ordering using interphase fluorescence *in situ* hybridization. Cytogenet Cell Genet 1993;64:49–53. 8508679 10.1159/000133559

[6] Nyberg P , Salo T, Kalluri R. Tumor microenvironment and angiogenesis. Front Biosci 2008;13:6537–53. 18508679 10.2741/3173

[7] Chen X , Song E. Turning foes to friends: targeting cancer-associated fibroblasts. Nat Rev Drug Discov 2019;18:99–115. 30470818 10.1038/s41573-018-0004-1

[8] Barrett RL , Puré E. Cancer-associated fibroblasts and their influence on tumor immunity and immunotherapy. Elife 2020;9:e57243. 33370234 10.7554/eLife.57243 PMC7769568

[9] Zhou Z , Guo S, Lai S, Wang T, Du Y, Deng J, . Integrated single-cell and bulk RNA sequencing analysis identifies a cancer-associated fibroblast-related gene signature for predicting survival and therapy in gastric cancer. BMC Cancer 2023;23:108. 36717783 10.1186/s12885-022-10332-w PMC9887891

[10] Ma H , Qiu Q, Tan D, Chen Q, Liu Y, Chen B, . The cancer-associated fibroblasts-related gene COMP is a novel predictor for prognosis and immunotherapy efficacy and is correlated with M2 macrophage infiltration in colon cancer. Biomolecules 2022;13:62. 36671447 10.3390/biom13010062 PMC9856124

[11] Elyada E , Bolisetty M, Laise P, Flynn WF, Courtois ET, Burkhart RA, . Cross-species single-cell analysis of pancreatic ductal adenocarcinoma reveals antigen-presenting cancer-associated fibroblasts. Cancer Discov 2019;9:1102–23. 31197017 10.1158/2159-8290.CD-19-0094 PMC6727976

[12] Chhabra Y , Weeraratna AT. Fibroblasts in cancer: unity in heterogeneity. Cell 2023;186:1580–609. 37059066 10.1016/j.cell.2023.03.016 PMC11422789

[13] Mao X , Xu J, Wang W, Liang C, Hua J, Liu J, . Crosstalk between cancer-associated fibroblasts and immune cells in the tumor microenvironment: new findings and future perspectives. Mol Cancer 2021;20:131. 34635121 10.1186/s12943-021-01428-1 PMC8504100

[14] Aggarwal S , Brennen WN, Kole TP, Schneider E, Topaloglu O, Yates M, . Fibroblast activation protein peptide substrates identified from human collagen I derived gelatin cleavage sites. Biochemistry 2008;47:1076–86. 18095711 10.1021/bi701921b PMC4696028

[15] Christiansen VJ , Jackson KW, Lee KN, McKee PA. Effect of fibroblast activation protein and alpha2-antiplasmin cleaving enzyme on collagen types I, III, and IV. Arch Biochem Biophys 2007;457:177–86. 17174263 10.1016/j.abb.2006.11.006 PMC1857293

[16] Xin L , Gao J, Zheng Z, Chen Y, Lv S, Zhao Z, . Fibroblast activation protein-alpha as a target in the bench-to-bedside diagnosis and treatment of tumors: a narrative review. Front Oncol 2021;11:648187. 34490078 10.3389/fonc.2021.648187 PMC8416977

[17] Brennen WN , Isaacs JT, Denmeade SR. Rationale behind targeting fibroblast activation protein-expressing carcinoma-associated fibroblasts as a novel chemotherapeutic strategy. Mol Cancer Ther 2012;11:257–66. 22323494 10.1158/1535-7163.MCT-11-0340 PMC3586189

[18] Brocks B , Garin-Chesa P, Behrle E, Park JE, Rettig WJ, Pfizenmaier K, . Species-crossreactive scFv against the tumor stroma marker “fibroblast activation protein” selected by phage display from an immunized FAP-/- knock-out mouse. Mol Med 2001;7:461–9. 11683371 PMC1950057

[19] Niedermeyer J , Garin-Chesa P, Kriz M, Hilberg F, Mueller E, Bamberger U, . Expression of the fibroblast activation protein during mouse embryo development. Int J Dev Biol 2001;45:445–7. 11330865

[20] Huang R , Pu Y, Huang S, Yang C, Yang F, Pu Y, . FAPI-PET/CT in cancer imaging: a potential novel molecule of the century. Front Oncol 2022;12:854658. 35692767 10.3389/fonc.2022.854658 PMC9174525

[21] Mori Y , Kratochwil C, Haberkorn U, Giesel FL. Fibroblast activation protein inhibitor theranostics: early clinical translation. PET Clin 2023;18:419–28. 37030981 10.1016/j.cpet.2023.02.007

[22] Galbiati A , Dorten P, Gilardoni E, Gierse F, Bocci M, Zana A, . Tumor-targeted interleukin 2 boosts the anticancer activity of FAP-directed radioligand therapeutics. J Nucl Med 2023;64:1934–40. 37734838 10.2967/jnumed.123.266007 PMC10690118

[23] Zana A , Puig-Moreno C, Bocci M, Gilardoni E, Di Nitto C, Principi L, . A comparative analysis of fibroblast activation protein-targeted small molecule-drug, antibody-drug, and peptide-drug conjugates. Bioconjug Chem 2023;34:1205–11. 37399501 10.1021/acs.bioconjchem.3c00244

[24] Poplawski SE , Hallett RM, Dornan MH, Novakowski KE, Pan S, Belanger AP, . Preclinical development of PNT6555, a boronic acid-based, fibroblast activation protein-alpha (FAP)-targeted radiotheranostic for imaging and treatment of FAP-positive tumors. J Nucl Med 2024;65:100–8. 38050111 10.2967/jnumed.123.266345

[25] LeBeau AM , Brennen WN, Aggarwal S, Denmeade SR. Targeting the cancer stroma with a fibroblast activation protein-activated promelittin protoxin. Mol Cancer Ther 2009;8:1378–86. 19417147 10.1158/1535-7163.MCT-08-1170 PMC3348578

[26] Brennen WN , Rosen DM, Wang H, Isaacs JT, Denmeade SR. Targeting carcinoma-associated fibroblasts within the tumor stroma with a fibroblast activation protein-activated prodrug. J Natl Cancer Inst 2012;104:1320–34. 22911669 10.1093/jnci/djs336 PMC3529592

[27] Hintz HM , Cowan AE, Shapovalova M, LeBeau AM. Development of a cross-reactive monoclonal antibody for detecting the tumor stroma. Bioconjug Chem 2019;30:1466–76. 30966746 10.1021/acs.bioconjchem.9b00206

[28] Hintz HM , Gallant JP, Vander Griend DJ, Coleman IM, Nelson PS, LeBeau AM. Imaging fibroblast activation protein alpha improves diagnosis of metastatic prostate cancer with positron emission tomography. Clin Cancer Res 2020;26:4882–91. 32636317 10.1158/1078-0432.CCR-20-1358 PMC7683011

[29] Dunaevsky YE , Tereshchenkova VF, Oppert B, Belozersky MA, Filippova IY, Elpidina EN. Human proline specific peptidases: a comprehensive analysis. Biochim Biophys Acta Gen Subj 2020;1864:129636. 32433934 10.1016/j.bbagen.2020.129636

[30] Rettig WJ , Su SL, Fortunato SR, Scanlan MJ, Raj BK, Garin-Chesa P, . Fibroblast activation protein: purification, epitope mapping and induction by growth factors. Int J Cancer 1994;58:385–92. 7519584 10.1002/ijc.2910580314

[31] Hintz HM , Snyder KM, Wu J, Hullsiek R, Dahlvang JD, Hart GT, . Simultaneous engagement of tumor and stroma targeting antibodies by engineered NK-92 cells expressing CD64 controls prostate cancer growth. Cancer Immunol Res 2021;9:1270–82. 34452926 10.1158/2326-6066.CIR-21-0178 PMC9119026

[32] Foss CA , Mease RC, Fan H, Wang Y, Ravert HT, Dannals RF, . Radiolabeled small-molecule ligands for prostate-specific membrane antigen: *in vivo* imaging in experimental models of prostate cancer. Clin Cancer Res 2005;11:4022–8. 15930336 10.1158/1078-0432.CCR-04-2690

[33] Miyahira AK , Soule HR. The history of prostate-specific membrane antigen as a theranostic target in prostate cancer: the cornerstone role of the prostate cancer foundation. J Nucl Med 2022;63:331–8. 34675109 10.2967/jnumed.121.262997

[34] Pandya DN , Sinha A, Yuan H, Mutkus L, Stumpf K, Marini FC, . Imaging of fibroblast activation protein alpha expression in a preclinical mouse model of glioma using positron emission tomography. Molecules 2020;25:3672. 32806623 10.3390/molecules25163672 PMC7464128

[35] Hsieh HH , Kuo WY, Lin JJ, Chen HS, Hsu HJ, Wu CY. Tumor-targeting ability of novel anti-prostate-specific membrane antigen antibodies. ACS Omega 2022;7:31529–37. 36092556 10.1021/acsomega.2c04230 PMC9454275

[36] Dumontet C , Reichert JM, Senter PD, Lambert JM, Beck A. Antibody-drug conjugates come of age in oncology. Nat Rev Drug Discov 2023;22:641–61. 37308581 10.1038/s41573-023-00709-2

[37] Sethakorn N , Heninger E, Breneman MT, Recchia E, Ding AB, Jarrard DF, . Integrated analysis of the tumor microenvironment using a reconfigurable microfluidic cell culture platform. FASEB J 2022;36:e22540. 36083096 10.1096/fj.202200684RR PMC9476232

[38] Saraon P , Cretu D, Musrap N, Karagiannis GS, Batruch I, Drabovich AP, . Quantitative proteomics reveals that enzymes of the ketogenic pathway are associated with prostate cancer progression. Mol Cell Proteomics 2013;12:1589–601. 23443136 10.1074/mcp.M112.023887 PMC3675816

[39] Vanli N , Guo-Fu HU. Mechanism and function of angiogenin in prostate cancer. Zhongguo Sheng Wu Hua Xue Yu Fen Zi Sheng Wu Xue Bao 2015;31:1261–6. 27175049 10.13865/j.cnki.cjbmb.2015.12.06 PMC4862603

[40] Li X , Liu Y, Wu B, Dong Z, Wang Y, Lu J, . Potential role of the OPG/RANK/RANKL axis in prostate cancer invasion and bone metastasis. Oncol Rep 2014;32:2605–11. 25333856 10.3892/or.2014.3511

[41] Mi J , Hooker E, Balog S, Zeng H, Johnson DT, He Y, . Activation of hepatocyte growth factor/MET signaling initiates oncogenic transformation and enhances tumor aggressiveness in the murine prostate. J Biol Chem 2018;293:20123–36. 30401749 10.1074/jbc.RA118.005395 PMC6311521

[42] Waugh DJ , Wilson C. The interleukin-8 pathway in cancer. Clin Cancer Res 2008;14:6735–41. 18980965 10.1158/1078-0432.CCR-07-4843

[43] Ara T , Declerck YA. Interleukin-6 in bone metastasis and cancer progression. Eur J Cancer 2010;46:1223–31. 20335016 10.1016/j.ejca.2010.02.026 PMC2917917

[44] Guo Y , Xu F, Lu T, Duan Z, Zhang Z. Interleukin-6 signaling pathway in targeted therapy for cancer. Cancer Treat Rev 2012;38:904–10. 22651903 10.1016/j.ctrv.2012.04.007

[45] Kalluri R . The biology and function of fibroblasts in cancer. Nat Rev Cancer 2016;16:582–98. 27550820 10.1038/nrc.2016.73

[46] Jung JE , Lee HG, Cho IH, Chung DH, Yoon SH, Yang YM, . STAT3 is a potential modulator of HIF-1-mediated VEGF expression in human renal carcinoma cells. FASEB J 2005;19:1296–8. 15919761 10.1096/fj.04-3099fje

[47] Li A , Dubey S, Varney ML, Dave BJ, Singh RK. IL-8 directly enhanced endothelial cell survival, proliferation, and matrix metalloproteinases production and regulated angiogenesis. J Immunol 2003;170:3369–76. 12626597 10.4049/jimmunol.170.6.3369

[48] Ashida Y , Okada M, Taniguchi I, Yamaga T. [An adult case of left ventricular-right atrial communication with a false aneurysm of membranous septum]. Nihon Kyobu Geka Gakkai Zasshi 1993;41:2431–4. 8288938

[49] Dong C . TH17 cells in development: an updated view of their molecular identity and genetic programming. Nat Rev Immunol 2008;8:337–48. 18408735 10.1038/nri2295

[50] Tanaka T , Narazaki M, Kishimoto T. IL-6 in inflammation, immunity, and disease. Cold Spring Harb Perspect Biol 2014;6:a016295. 25190079 10.1101/cshperspect.a016295 PMC4176007

[51] Jones CV , Ricardo SD. Macrophages and CSF-1: implications for development and beyond. Organogenesis 2013;9:249–60. 23974218 10.4161/org.25676 PMC3903694

[52] Gschwandtner M , Derler R, Midwood KS. More than just attractive: how CCL2 influences myeloid cell behavior beyond chemotaxis. Front Immunol 2019;10:2759. 31921102 10.3389/fimmu.2019.02759 PMC6923224

[53] Cha JH , Chan LC, Li CW, Hsu JL, Hung MC. Mechanisms controlling PD-L1 expression in cancer. Mol Cell 2019;76:359–70. 31668929 10.1016/j.molcel.2019.09.030 PMC6981282

[54] Hensbergen PJ , Wijnands PG, Schreurs MW, Scheper RJ, Willemze R, Tensen CP. The CXCR3 targeting chemokine CXCL11 has potent antitumor activity *in vivo* involving attraction of CD8+ T lymphocytes but not inhibition of angiogenesis. J Immunother 2005;28:343–51. 16000952 10.1097/01.cji.0000165355.26795.27

[55] Bonollo F , Thalmann GN, Kruithof-de Julio M, Karkampouna S. The role of cancer-associated fibroblasts in prostate cancer tumorigenesis. Cancers 2020;12:1887. 32668821 10.3390/cancers12071887 PMC7409163

[56] Garin-Chesa P , Old LJ, Rettig WJ. Cell surface glycoprotein of reactive stromal fibroblasts as a potential antibody target in human epithelial cancers. Proc Natl Acad Sci U S A 1990;87:7235–9. 2402505 10.1073/pnas.87.18.7235 PMC54718

[57] Rettig WJ , Garin-Chesa P, Healey JH, Su SL, Ozer HL, Schwab M, . Regulation and heteromeric structure of the fibroblast activation protein in normal and transformed cells of mesenchymal and neuroectodermal origin. Cancer Res 1993;53:3327–35. 8391923

[58] Zou B , Liu X, Zhang B, Gong Y, Cai C, Li P, . The expression of FAP in hepatocellular carcinoma cells is induced by hypoxia and correlates with poor clinical outcomes. J Cancer 2018;9:3278–86. 30271487 10.7150/jca.25775 PMC6160687

[59] Wikberg ML , Edin S, Lundberg IV, Van Guelpen B, Dahlin AM, Rutegard J, . High intratumoral expression of fibroblast activation protein (FAP) in colon cancer is associated with poorer patient prognosis. Tumour Biol 2013;34:1013–20. 23328994 10.1007/s13277-012-0638-2 PMC3597266

[60] Cohen SJ , Alpaugh RK, Palazzo I, Meropol NJ, Rogatko A, Xu Z, . Fibroblast activation protein and its relationship to clinical outcome in pancreatic adenocarcinoma. Pancreas 2008;37:154–8. 18665076 10.1097/MPA.0b013e31816618ce

[61] Haffner MC , Zwart W, Roudier MP, True LD, Nelson WG, Epstein JI, . Genomic and phenotypic heterogeneity in prostate cancer. Nat Rev Urol 2021;18:79–92. 33328650 10.1038/s41585-020-00400-w PMC7969494

[62] Beltran H , Prandi D, Mosquera JM, Benelli M, Puca L, Cyrta J, . Divergent clonal evolution of castration-resistant neuroendocrine prostate cancer. Nat Med 2016;22:298–305. 26855148 10.1038/nm.4045 PMC4777652

[63] Glumac PM , Gallant JP, Shapovalova M, Li Y, Murugan P, Gupta S, . Exploitation of CD133 for the targeted imaging of lethal prostate cancer. Clin Cancer Res 2020;26:1054–64. 31732520 10.1158/1078-0432.CCR-19-1659 PMC7056526

[64] Vlachostergios PJ , Karathanasis A, Tzortzis V. Expression of fibroblast activation protein is enriched in neuroendocrine prostate cancer and predicts worse survival. Genes 2022;13:135. 35052475 10.3390/genes13010135 PMC8774973

[65] Goldstein LA , Ghersi G, Pineiro-Sanchez ML, Salamone M, Yeh Y, Flessate D, . Molecular cloning of seprase: a serine integral membrane protease from human melanoma. Biochim Biophys Acta 1997;1361:11–9. 9247085 10.1016/s0925-4439(97)00032-x

[66] Narra K , Mullins SR, Lee HO, Strzemkowski-Brun B, Magalong K, Christiansen VJ, . Phase II trial of single agent Val-boroPro (Talabostat) inhibiting fibroblast activation protein in patients with metastatic colorectal cancer. Cancer Biol Ther 2007;6:1691–9. 18032930 10.4161/cbt.6.11.4874

[67] Zboralski D , Hoehne A, Bredenbeck A, Schumann A, Nguyen M, Schneider E, . Preclinical evaluation of FAP-2286 for fibroblast activation protein targeted radionuclide imaging and therapy. Eur J Nucl Med Mol Imaging 2022;49:3651–67. 35608703 10.1007/s00259-022-05842-5 PMC9399058

[68] Hofheinz RD , al-Batran SE, Hartmann F, Hartung G, Jager D, Renner C, . Stromal antigen targeting by a humanised monoclonal antibody: an early phase II trial of sibrotuzumab in patients with metastatic colorectal cancer. Onkologie 2003;26:44–8. 12624517 10.1159/000069863

[69] Loktev A , Lindner T, Burger EM, Altmann A, Giesel F, Kratochwil C, . Development of fibroblast activation protein-targeted radiotracers with improved tumor retention. J Nucl Med 2019;60:1421–9. 30850501 10.2967/jnumed.118.224469 PMC6785792

[70] Kobayashi T , Noma K, Nishimura S, Kato T, Nishiwaki N, Ohara T, . Near-infrared photoimmunotherapy targeting cancer-associated fibroblasts in patient-derived xenografts using a humanized anti-fibroblast activation protein antibody. Mol Cancer Ther 2024 [Online ahead of print]. 10.1158/1535-7163.MCT-23-0527 38638034

[71] D'Amico L , Menzel U, Prummer M, Muller P, Buchi M, Kashyap A, . A novel anti-HER2 anthracycline-based antibody-drug conjugate induces adaptive anti-tumor immunity and potentiates PD-1 blockade in breast cancer. J Immunother Cancer 2019;7:16. 30665463 10.1186/s40425-018-0464-1 PMC6341578

[72] Tiberghien AC , Levy JN, Masterson LA, Patel NV, Adams LR, Corbett S, . Design and synthesis of tesirine, a clinical antibody-drug conjugate pyrrolobenzodiazepine dimer payload. ACS Med Chem Lett 2016;7:983–7. 27882195 10.1021/acsmedchemlett.6b00062 PMC5108040

[73] Sung KE , Su X, Berthier E, Pehlke C, Friedl A, Beebe DJ. Understanding the impact of 2D and 3D fibroblast cultures on in vitro breast cancer models. PLoS One 2013;8:e76373. 24124550 10.1371/journal.pone.0076373 PMC3790689

