# Supplementary material for: Mechanistic Characterization of Cancer-associated Fibroblast Depletion via an Antibody–Drug Conjugate Targeting Fibroblast Activation Protein
Source: Cancer Res Commun. 2024 Jun 12;4(6):1481–94. doi: 10.1158/2767-9764.CRC-24-0248 (PMC11168342; doi:10.1158/2767-9764.CRC-24-0248)
Supplement: Supplementary Figure 1 — Antibody Humanization [file crc-24-0248-s01.pdf]

Supplemental Data

Supplemental Figure 1. Humanization was performed by Fusion Antibodies PLC, Belfast, UK. The binding domains of our anti-FAP antibody B12 were identified from a murine single-chain variable fragment phage display library. For humanization, the heavy and light chains CDRs of B12 were identified using the antibody numbering systems from IMGT and Kabat. Human framework sequences were identified and used as acceptor frameworks for the B12 CDR sequences. The acceptor sequences were from a mature human IgG1 to ensure that the humanized sequences were non-immunogenic and retained the canonical structure of the CDR loops. The amino acid sequences of the humanized variants were found to be more similar to human variable region than any other species, indicating humanization. HC7LC10 (huB12) was selected because its properties closely mirrored parental B12.

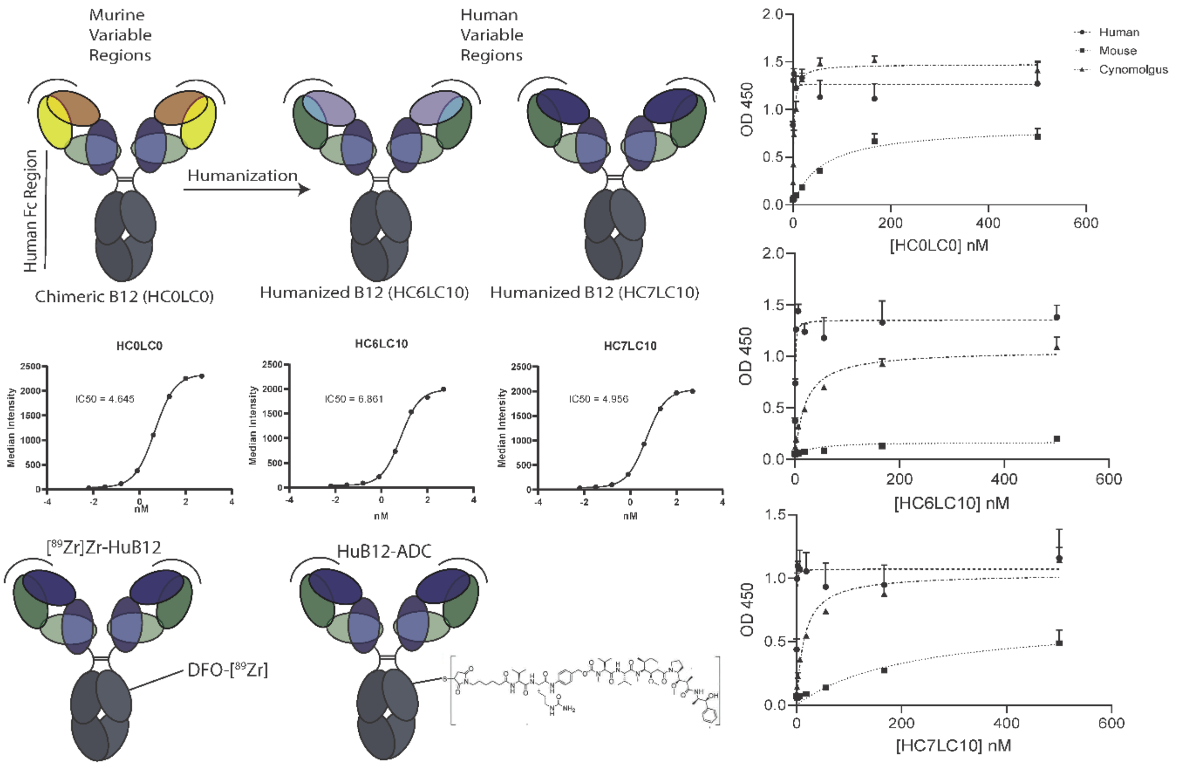

Parental B12 (H0LC0)

H0

QVQLVESGGGLVQPGGSLKLSCAASGFTFSSYGMSWVRQTPDKRLELVATINSNGGSTYYPD
SVKGRFTISRDNAKNTLYLQMSSLKSEDTAMYYCARDYFDYWGQGTTLTVSS

LC0

DIVITQSPSSLSASLGERVSLTCRASQEISGYLSWLQQKPDGTIKRLIYAASTLDSGVPKRFSGS
RSGSDYSLTISSLESEDFADYYCLQYASYPWTFGGGTKLEIK

huB12 (HC7LC10)

HC7

QVQLVESGGGLVQPGGSLRLSCAASGFTFSSYGMSWVRQAPGKKLELVATINSNGGSTYYPD
SVKGRFTISRDNSKNTLYLQMNSLRAEDTAVYYCARDYFDYWGQGTTLTVSS

LC10

DIQMTQSPSSLSASVGDRVTITCRASQEISGYLSWLQQKPEGTIKRLIYAASSTLDSGVPSRFSG
SGSGTDFTLTISSLQPEDFADYYCLQYASYPWTFGGGTKLEIK
