# Supplementary material for: Mechanistic Characterization of Cancer-associated Fibroblast Depletion via an Antibody–Drug Conjugate Targeting Fibroblast Activation Protein
Source: Cancer Res Commun. 2024 Jun 12;4(6):1481–94. doi: 10.1158/2767-9764.CRC-24-0248 (PMC11168342; doi:10.1158/2767-9764.CRC-24-0248)

Supplemental Figure 2. Therapeutic efficacy of the three huB12 ADCs with B12 and D8-MMAE as controls in five different cell lines: R1enzR(FAP^{null}), R1enzR-FAP(FAP^{pos}), MDA-MB-436(FAP^{pos}), and SK-MEL-187(FAP^{pos}). Experimental protocol is described in Materials and Methods.

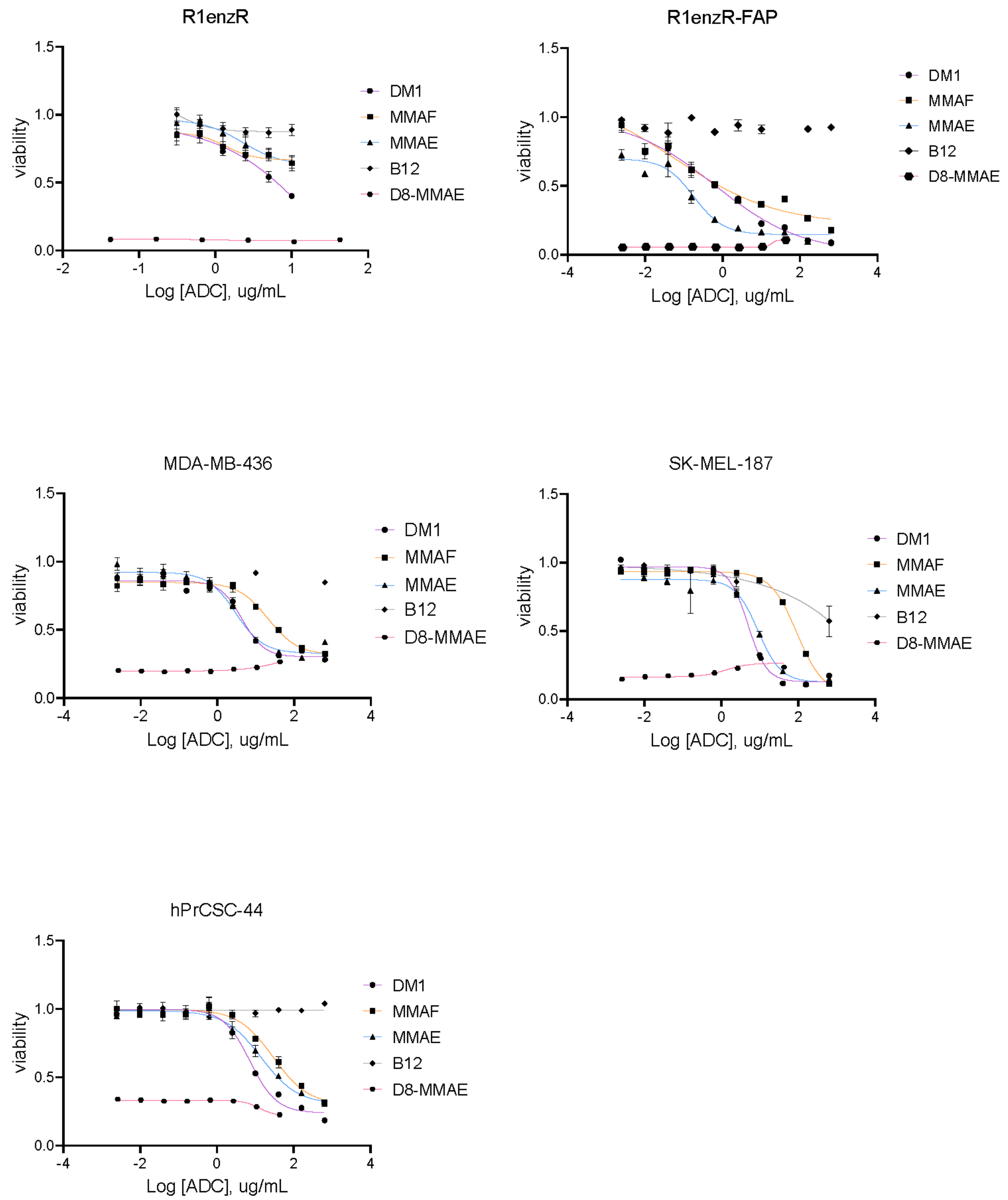

Supplement: Supplementary Figure 2 — ADC efficacy in multiple cell lines [file crc-24-0248-s02.pdf]
