# Supplementary material for: Mechanistic Characterization of Cancer-associated Fibroblast Depletion via an Antibody–Drug Conjugate Targeting Fibroblast Activation Protein
Source: Cancer Res Commun. 2024 Jun 12;4(6):1481–94. doi: 10.1158/2767-9764.CRC-24-0248 (PMC11168342; doi:10.1158/2767-9764.CRC-24-0248)

Supplemental Figure 3. Determining the therapeutic window of hPrCSC-44 cells treated with huB12-MMAE. Monoculture hPrCSC-44 cells were treated in the Stack with huB12-MMAE at seven different concentrations for 72h. Cell viability was determined by Calcein-AM fluorescent intensity as described in the Materials and Methods section. Assays were performed in triplicate a total of five times. Values represent mean ± SEM. *, P ≤ 0.05.

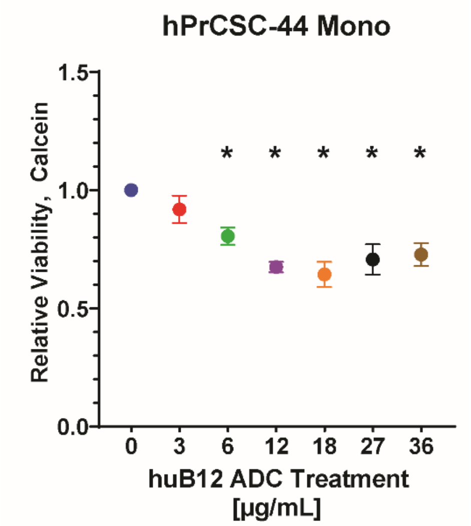

Supplement: Supplementary Figure 3 — Determining the therapeutic window in the Stacks [file crc-24-0248-s03.pdf]
