# Supplementary material for: Mechanistic Characterization of Cancer-associated Fibroblast Depletion via an Antibody–Drug Conjugate Targeting Fibroblast Activation Protein
Source: Cancer Res Commun. 2024 Jun 12;4(6):1481–94. doi: 10.1158/2767-9764.CRC-24-0248 (PMC11168342; doi:10.1158/2767-9764.CRC-24-0248)

Supplemental Figure 5. mRNA expression of VEGFA under different treatment conditions in 22Rv1 cells (left) and hPrCSC-44 cells (right). The protocol and TaqMan primers used are described in the Materials and Methods section. Assays were performed in triplicate from five experimental replicates. Values represent mean ± SEM. *, P ≤ 0.05.

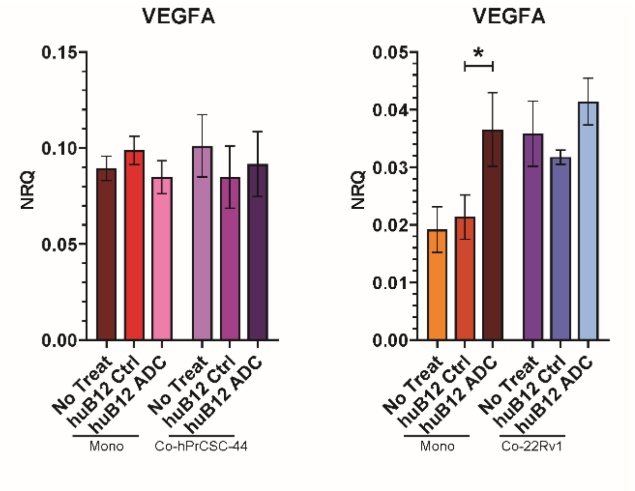

Supplement: Supplementary Figure 5 — VEGFA expression [file crc-24-0248-s05.pdf]
