# Supplementary material for: Mechanistic Characterization of Cancer-associated Fibroblast Depletion via an Antibody–Drug Conjugate Targeting Fibroblast Activation Protein
Source: Cancer Res Commun. 2024 Jun 12;4(6):1481–94. doi: 10.1158/2767-9764.CRC-24-0248 (PMC11168342; doi:10.1158/2767-9764.CRC-24-0248)

Supplemental Figure 6. mRNA expression data of 22Rv1 cells and hPrCSC-44 cells under different treatment and culture conditions. **A**) Expression of pro-inflammatory cytokines in 22Rv1 cells. **B**) Expression of known immunosuppressive genes in 22Rv1 cells. **C**) Expression of select cytokines in hPrCSC-44 cells. The protocol and TaqMan primers used are described in the Materials and Methods section. Assays were performed in triplicate from five experimental repeats. Values represent mean ± SEM. *, P ≤ 0.05.

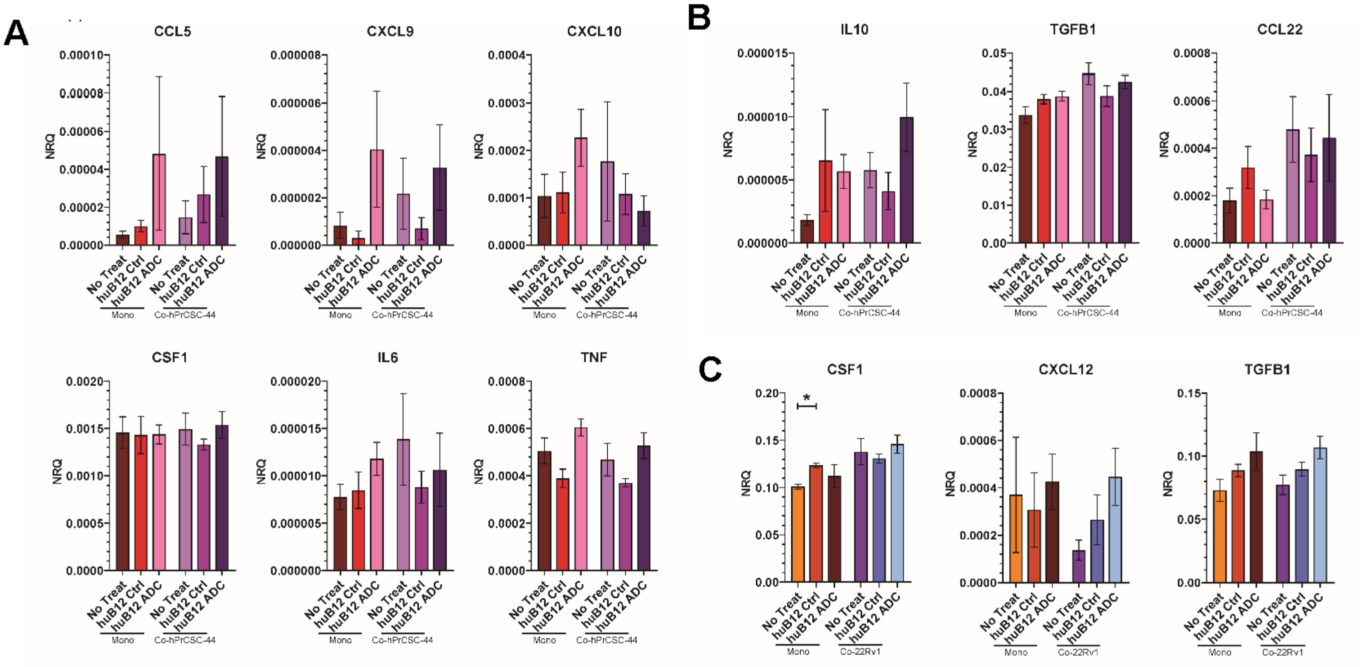

Supplement: Supplementary Figure 6 — mRNA expression of cytokines [file crc-24-0248-s06.pdf]
